# Interface-Engineered ZIF-8-Derived ZnO@C/Bi/Bi_2_MoO_6_ Heterojunctions for Boosting Photo-Fenton-like Performance

**DOI:** 10.3390/nano16211393

**Published:** 2026-10-29

**Authors:** Yixuan Hu, Jun Ke, Zhaoyang Yan, Jie Liu

**Affiliations:** 1 Department of Environmental Science and Engineering, North China Electric Power University, Baoding 071003, China; 2 MOE Key Laboratory of Resources and Environmental Systems Optimization, North China Electric Power University, Beijing 102206, China

**Keywords:** carbon materials, MOF derived, tetracycline degradation, interfacial charge transfer

## Abstract

In this work, a novel ZnO@C/Bi/Bi_2_MoO_6_ heterojunction is synthesized by assembling bismuth molybdate (Bi_2_MoO_6_) and ZIF-8-derived ZnO@C through a hydrothermal method for enhancing the photo-Fenton-like performance, where a tight interface contact is efficiently formed. The heterojunction not only improves the harvesting ability to visible light, but also promotes the separation and transfer of photogenerated electrons and holes. As a result, BZ400-2 achieves 69.8% degradation of tetracycline (TC) within 90 min under visible light irradiation (300 W Xe lamp; λ > 420 nm), with an apparent pseudo-first-order rate constant of 0.01081 min^−1^. Meanwhile, radical quenching experiments and ESR measurements confirm the involvement of·O_2_^−^, ^1^O_2_ and ·OH in the reaction system. Furthermore, the results of DFT theory demonstrate that the phenolic hydroxyl, ketone, and dimethylamine groups of tetracycline hydrochloride are more susceptible to free radical attack. Furthermore, the QSAR-based toxicity predictions suggest that most of the identified degradation intermediates exhibit lower acute toxicity than TC.

## 1. Introduction

The Fenton reaction, first reported by Henry J. Fenton in 1894, involves the activation of hydrogen peroxide (H_2_O_2_) by ferrous ions (Fe^2+^) to generate hydroxyl radicals (·OH). However, regeneration of Fe^3+^ into Fe^2+^ and formation of iron sludge are the main limiting factors to the wide application of Fenton reaction [1]. To overcome these challenges, heterogeneous photo-Fenton processes have been developed; in these heterogeneous photo-Fenton-like systems, the activation of H_2_O_2_ is facilitated by surface-catalyzed reactions or photogenerated charge carriers [2,3,4]. More importantly, the low-valent metal ions (Co^2+^, Cu^+^, Mn^2+^, etc.) in the catalysts can be regenerated through the reduction of high-valent metal ions (Co^3+^ [5], Cu^2+^ [6], Mn^4+^ [7], etc.) by the photoinduced electrons under light illumination. Furthermore, the heterogeneous photo-Fenton process is more efficient than the homogeneous one, because these catalysts can provide a large surface area for reaction with a low concentration of catalyst [8]. Consequently, the photo-Fenton system can function efficiently from near-neutral to alkaline pH conditions, which provides a distinct advantage over the traditional Fenton reaction. For example, Cu-based catalysts activate H_2_O_2_ at near-neutral pH through the Cu^+^/Cu^2+^ redox cycle [9], while Co- and Mn-based systems rely on multivalent redox and oxygen vacancy-mediated pathways that can be enhanced under visible light [10,11]. In contrast, a Ce-based catalyst with redox of Ce^3+^/Ce^4+^ can facilitate H_2_O_2_ activation primarily via photogenerated charge carriers with oxygen vacancies [12] or surface plasmon resonance-assisted interfacial electron transfer [13].

Recently, bismuth-based materials have emerged as a highly promising class of non-ferrous photo-Fenton-like catalysts [14], which possesses unique internal electric fields (IEFs) and facilitates rapid charge separation, while their tunable band structures allow for strong light absorption and high ROS generation [15,16]. For example, An et al. attempted to integrate photocatalysis and Fenton-like catalysis by using BiFeO_3_ as a heterogeneous photocatalyst to improve the decomposition of methyl violet under visible light irradiation [17].

To further improve the catalytic performance of bismuth-based materials, various strategies have been proposed, including element doping, morphology control, interface engineering, and heterojunction construction [18]. Among them, by associating Bi_2_MoO_6_ with other semiconductors, a novel heterojunction structure can be created to realize the separation and transfer of photogenerated charge carriers, thereby improving the efficiency of the photo-Fenton degradation process [19,20]. Recently, metal–organic frameworks (MOFs) have been developed as a Fenton-like process, due to the high specific surface area, controllable pore size and highly exposed active metal sites [21,22]. For instance, Bai et al. reported a nano-interfacial BiFeVO_4_/NH_2_-MIL-88B with abundant vacancy defects for efficient photo-Fenton-like degradation of oxytetracycline [23]. The nano-interfacial composite displayed enhanced electrical conductivity, broadened light absorption and a narrowed band gap. More interestingly, as precursors or sacrificial templates, MOFs can be used to generate porous carbon-based composites due to their unique engineering morphology and high-crystallinity matrix materials [24,25]. For example, Huang et al. fabricated MOF-derived Fe_2_O_3_@C-coupled Bi_2_MoO_6_ heterojunctions that achieved 93.2% tetracycline decomposition in a photo-Fenton system induced by the synergistic effect of Fenton oxidation and photocatalysis [26]. Moreover, Qian et al. prepared ZnO@FePc photo-Fenton catalysts by using ZIF-8 as a precursor, where a high-speed electron transmission channel was constructed through surface hydroxyl-induced assembly [27], which achieved 90% ibuprofen degradation within 10 min under simulated sunlight. Therefore, it is expected that coupling Bi_2_MoO_6_ with an MOF-derived metal oxide@carbon composite could efficiently improve the separation and transfer of photogenerated charge carriers with high harvesting capacity of sunlight.

Furthermore, efficient H_2_O_2_ activation remains a key challenge in heterogeneous Fenton-like systems. Traditional Fenton and Fenton-like processes, which employ variable-valence metals, such as Fe, Ce, and Co, are plagued by issues including sludge generation, high costs, and heavy metal contamination; consequently, exploring novel pathways for hydrogen peroxide activation has become a new research direction. Ran et al. synthesized Bi_2_WO_6_ nanostructures with a controlled morphology to serve as photocatalytic-Fenton catalysts for the treatment of pulp mill wastewater; under ultraviolet irradiation, the catalyst achieved a chemical oxygen demand (CODCr) removal efficiency of 85.8% [28]. However, Ran et al. did not conduct an in-depth investigation into the mechanism of hydrogen peroxide activation within this system.

**Scheme 1:**
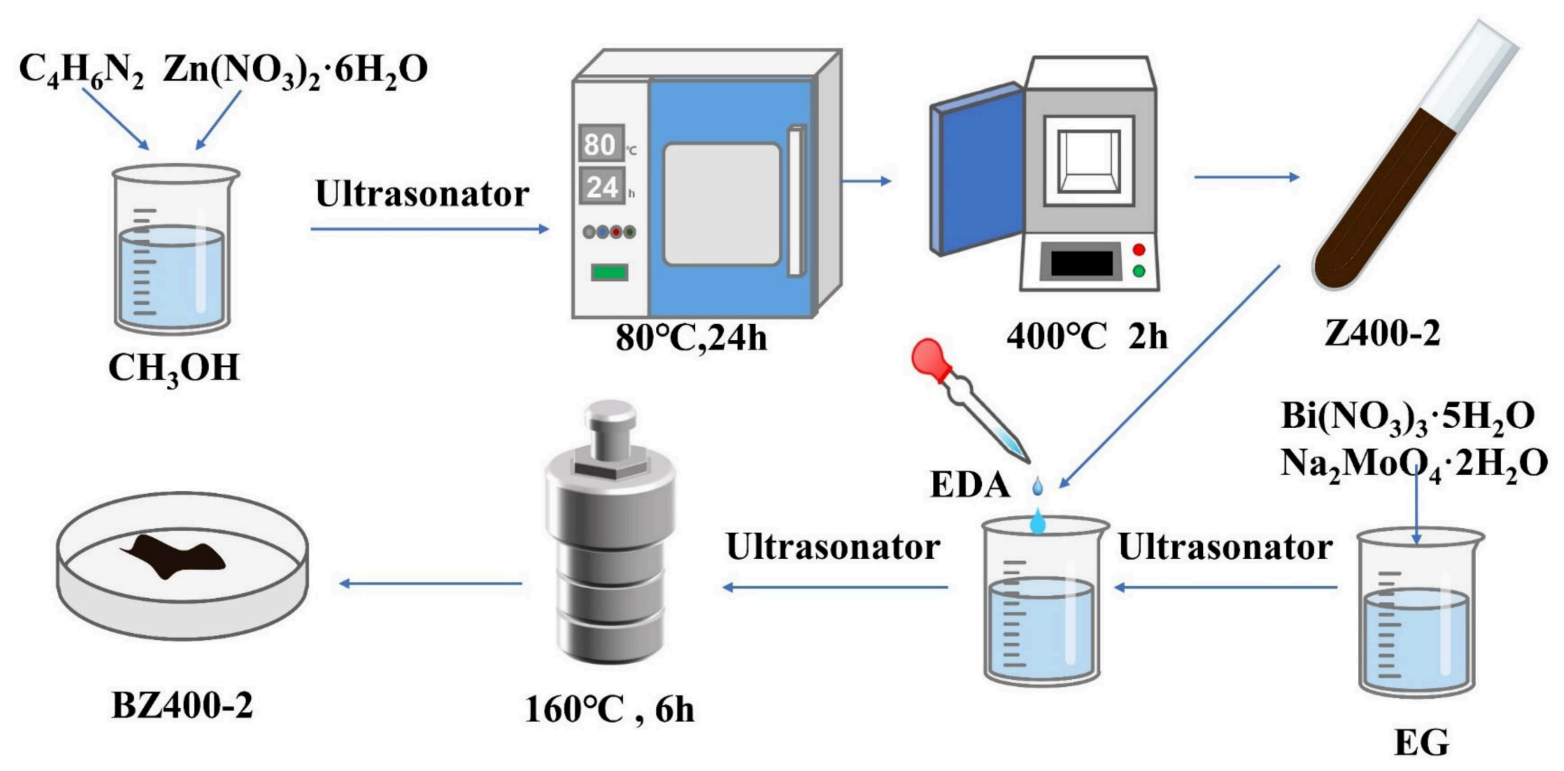
Synthesis path of ZIF-derived ZnO@C/Bi/Bi_2_MoO_6_ plasmonic heterojunctions.

Based on the aforementioned approaches, this study constructed a novel ZnO@C/Bi/Bi_2_MoO_6_ heterojunction (Scheme 1) by integrating ZnO@C with plasmonic Bi/Bi_2_MoO_6_ via a one-step hydrothermal method, offering a new perspective on the application of MOF-derived materials in Fenton-like reactions. First, ZnO@C was prepared by calcining ZIF-8 as a precursor. The phase structure, morphology, surface valence states, and photoelectrochemical properties of the resulting samples were subsequently characterized and analyzed. The study revealed the formation of intimate interfacial contact between ZnO@C and Bi/Bi_2_MoO_6_, which not only enhanced the visible light harvesting capabilities but also significantly improved the separation efficiency of photogenerated electron-hole pairs, suppressing electron-hole recombination and facilitating electron transfer to adsorbed H_2_O_2_ for activation; this enhanced the degradation efficiency of tetracycline (TC) by the photo-Fenton-like system under visible light irradiation. Furthermore, the degradation mechanism of TC was investigated through a combination of radical trapping experiments and density functional theory (DFT) calculations.

## 2. Experimental Section

### 2.1. Materials

Bismuth nitrate pentahydrate (Bi(NO_3_)_3_·5H_2_O), sodium molybdate dihydrate (Na_2_MoO_4_·2H_2_O), zinc nitrate hexahydrate (Zn(NO_3_)_2_·6H_2_O), 2-methylimidazole (C_4_H_6_N_2_), ethylene glycol (C_2_H_6_O_2_), ethylenediamine (C_2_H_8_N_2_), methanol (CH_3_OH), anhydrous ethanol (C_2_H_6_O) were purchased from Sinopharm Chemical Reagent Co., Ltd. Tetracycline hydrochloride (TC) was purchased from Merck Co., Ltd. Hydrogen peroxide (H_2_O_2_, 30 wt%), sodium sulfate (Na_2_SO_4_), hydrochloric acid (HCl), sodium hydroxide (NaOH), sodium oxalate (Na_2_C_2_O_4_), tert-butanol (TBA), L-histidine (L-His), silver nitrate (AgNO_3_), and ascorbic acid (VC) were purchased from Tianjin Kermel Chemical Reagent Co., Ltd. in this study. All reagents were of analytical grade and were used without further purification.

### 2.2. Synthesis of ZIF-8 Precursor

In a typical procedure, 10 mmol of zinc nitrate hexahydrate and 80 mmol of 2-methylimidazole were added into 100 mL of methanol, and stirred vigorously at room temperature for 1 h. The product was filtered, washed twice with methanol, and dried at 80 °C for 24 h, and was named ZIF-8.

### 2.3. Synthesis of ZnO@C Composites

The ZIF-8 precursor was placed in a muffle furnace and calcined at 400 °C for 2 h under an air atmosphere, with a heating rate of 5 °C/min. The obtained sample was denoted as Z400-2. To investigate effects of calcination temperature, samples were synthesized at 300 °C, 400 °C and 500 °C, and named Z300-2, Z400-2 and Z500-2, respectively.

### 2.4. Synthesis of ZnO@C/Bi/Bi_2_MoO_6_ Heterojunction

Firstly, 1.0 mmol of Bi(NO_3_)_3_·5H_2_O and 0.5 mmol of Na_2_MoO_4_·2H_2_O were added into 15 mL of ethylene glycol and sonicated until homogeneous and transparent. Subsequently, 0.3 mL of ethylenediamine and ZnO@C (20–50 wt% relative to the mass of Bi(NO_3_)_3_·5H_2_O) were added to the above solution. After sonication for 30 min, the mixture was transferred to a stainless-steel high-pressure reactor with a polytetrafluoroethylene liner and heated in an oven at 160 °C for 6 h. After cooling, the precipitate was collected by centrifugation, washed several times alternately with deionized water and ethanol, and finally dried at 70 °C for 12 h. The materials were designated as BZ20, BZ30, and BZ50 based on the varying amounts of Z400-2 incorporated; additionally, a pure-phase Bi_2_MoO_6_ sample was prepared without ZnO@C doping. Depending on the calcination temperature (℃) and duration (h) of the ZIF-8 precursor, the resulting composite materials were labeled as BZ℃-h.

### 2.5. Photoelectrochemical Testing

Electrochemical measurements were conducted on a CHI660E electrochemical workstation (Chenhua, China) coupled with a xenon lamp as the light source. A conventional three-electrode system was employed, consisting of a catalyst-coated FTO glass working electrode, a platinum wire counter electrode, and an Ag/AgCl reference electrode. The working electrode was prepared by depositing the catalyst onto FTO glass using a 1:1 (*v*/*v*) mixture of ethanol and turpentine. A 0.1 mol/L Na_2_SO_4_ aqueous solution was used as the supporting electrolyte. Transient photocurrent measurements were performed at an applied potential of 0.27 V vs. Ag/AgCl under irradiation from a 300 W xenon lamp with a light intensity of 100 mW/cm^2^. The transient photocurrent responses (i-t), electrochemical impedance spectroscopy (EIS), and Mott–Schottky measurements were performed to evaluate the photoelectrochemical properties of the prepared samples. The Mott–Schottky plots were recorded at frequencies of 500, 800, and 1000 Hz in 0.1 mol L^−1^ Na_2_SO_4_ electrolyte with a measured pH of 7.

### 2.6. Photo-Fenton Performance Testing

In the typical photo-Fenton experiment, 10 mg of the as-prepared catalyst was dispersed into 30 mL of tetracycline solution (50 mg/L). The initial pH of the reaction solution under standard experimental conditions was approximately 4.3 without additional pH adjustment. Prior to irradiation, the suspension was stirred in the dark for 30 min to establish adsorption–desorption equilibrium. Subsequently, the suspension was exposed to a 300 W Xe lamp (100 mW/cm^2^) equipped with a 420 nm cut-off filter. At the onset of illumination, 10 μL of H_2_O_2_ (30 wt%) was introduced into the reaction system. All photo-Fenton-like degradation experiments were conducted at room temperature. At given time intervals, 1 mL of solution was withdrawn using a syringe, immediately filtered through a 0.22 μm membrane to remove the catalyst, and the resulting filtrate was collected for analysis. The TC concentration in the filtrate was determined by high-performance liquid chromatography (HPLC, Thermo scientific).

## 3. Results and Discussion

**Figure 1:**
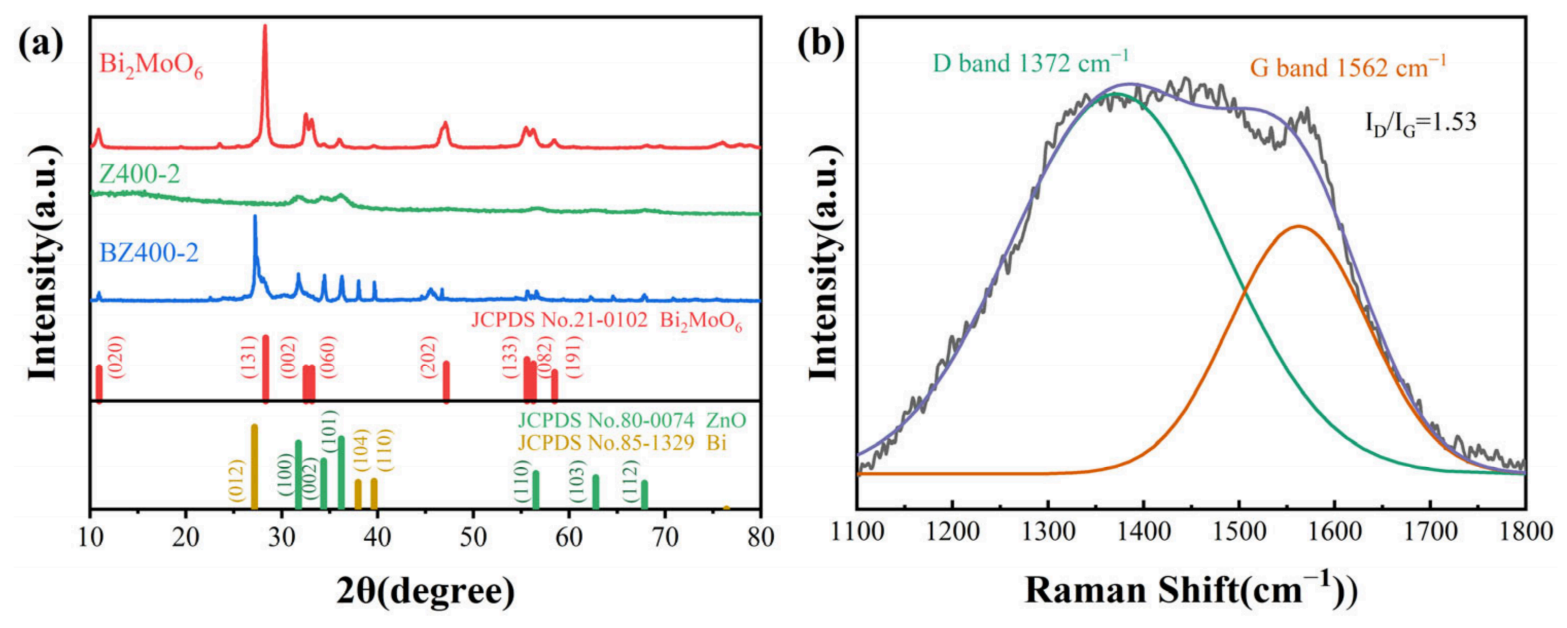
(**a**) XRD patterns of Bi_2_MoO_6_, Z400-2 and BZ400-2 composites; (**b**) Raman spectra of the prepared BZ400-2 heterojunction.

The crystal structures of the ZIF-8 derivatives and the composite heterojunction were analyzed using X-ray diffraction (XRD, SmartLab SE, Japan). As shown in Figure S1a, Z300-2 retained the characteristic diffraction peaks of ZIF-8, indicating that the ZIF-8 framework was not completely transformed at 300 °C. When the calcination temperature reached 400 °C, the organic framework of ZIF-8 was gradually destroyed, and characteristic peaks of ZnO appeared, where the characteristic peaks at 31.7°, 34.4°, 36.2°, 56.5°, 62.8°, and 67.9° correspond to the (100), (002), (101), (110), (103), and (112) crystal planes of hexagonal ZnO (JCPDS No. 80-0074), respectively [29]. As the calcination temperature rose to 500 °C, the characteristic peaks of ZnO became more pronounced; there is a possibility of carbon loss due to oxidation in the air. In Figure 1a, it can be seen that the diffraction peaks of Bi_2_MoO_6_ appear at 10.93°, 28.31°, 32.64°, 33.14°, 46.74°, 55.89°, 56.40° and 58.47°, which are attributed to the (020), (131), (002), (060), (202), (133), (082) and (191) planes of orthorhombic Bi_2_MoO_6_ (JCPDS No. 21-0102) [30]. After coupling Z400-2 with Bi_2_MoO_6_, the characteristic diffraction peaks of Bi_2_MoO_6_ remain clearly observable in the BZ400-2 composite, confirming the successful formation of the heterojunction without destroying the crystal structure of Bi_2_MoO_6_. The diffraction peaks at 32.64°, 55.89° and 56.40° correspond to the (002) crystal plane of Bi_2_MoO_6_. The characteristic peaks at 31.73°, 34.36°, and 36.21° correspond to the (100), (002), and (101) crystal planes of ZnO, respectively, indicating that Bi_2_MoO_6_ and ZnO coexist in the heterojunction. In addition, new characteristic peaks at 27.16°, 37.95°, and 39.62° were detected, which are attributed to the (012), (104), and (110) crystal planes of metallic Bi (JCPDS No. 85-1329) [31], indicating that some Bi_2_MoO_6_ was reduced to metallic Bi during the hydrothermal synthesis process. Analysis of the reaction process suggests that the carbon generated during the calcination of ZIF-8 possesses reducing capabilities, enabling the reduction of some Bi^3+^ ions into metallic Bi particles [32]. Figure 1b shows the Raman spectrum of BZ400-2, where the peaks at 1372 cm^−1^ and 1562 cm^−1^ correspond to the D and G bands of carbon, respectively. The calculated peak-height intensity ratio (I_D_/I_G_) is 1.53, suggesting the presence of structural disorder in the carbon component [33]. These results, together with the XPS analysis, support the presence of carbonaceous species in BZ400-2. The carbon component may facilitate electron transport, thereby promoting the separation and transfer of photogenerated charge carriers.

**Figure 2:**
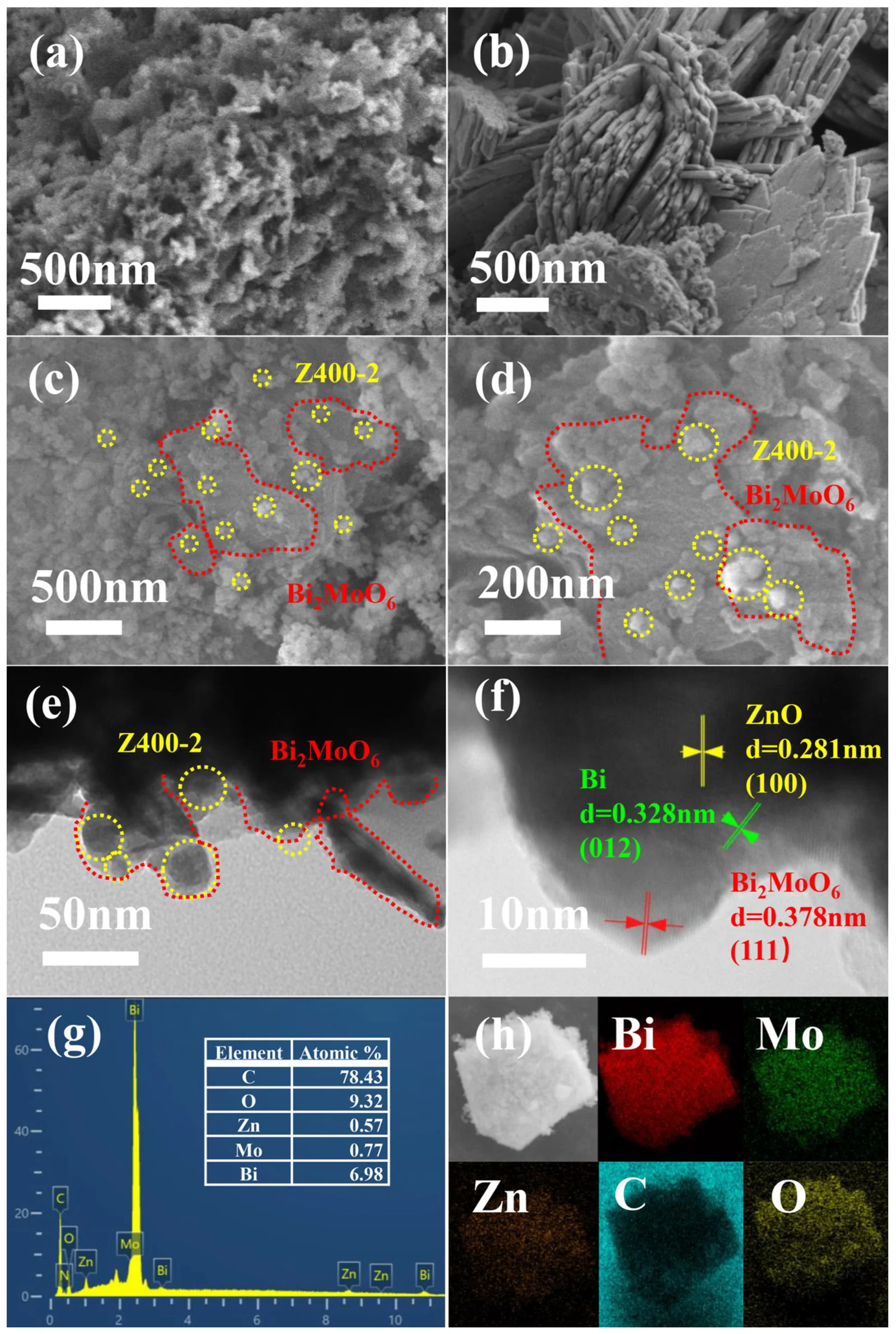
SEM images of (**a**) Z400-2, (**b**) Bi_2_MoO_6_, and (**c**,**d**) BZ400-2; (**e**) TEM image; (**f**) HRTEM image; (**g**) EDS spectrum; and (**h**) elemental mapping images of Bi, Mo, Zn, C, and O in BZ400-2.

The morphologies of Z400-2, Bi_2_MoO_6_ and BZ400-2 are displayed in Figure 2. As shown in Figure 2a, after calcination at 400 °C for 2 h, Z400-2 exhibits coral-like aggregates of small particles, with an average particle size of approximately 54 nm. Figure 2b shows that the pure Bi_2_MoO_6_ presents a typical layered structure [34]. The morphologies of the BZ400-2 composite are displayed in Figure 2c,d. It was found that after the hydrothermal treatment, the Z400-2 nanoparticles were deposited onto the surface of Bi_2_MoO_6_ nanosheets, verifying the tight contact between ZnO@C and Bi_2_MoO_6_. The prepared BZ400-2 was characterized by transmission electron microscopy (TEM). Figure 2e shows that the Z400-2 nanoparticles are tightly attached to the Bi_2_MoO_6_ nanosheets, which is beneficial for the formation of heterojunctions. As shown in Figure 2f, lattice spacings of approximately 0.378, 0.328, and 0.281 nm are observed. The spacing of 0.281 nm corresponds to the (100) plane of ZnO, while the spacings of 0.378 nm and 0.328 nm can be attributed to the (111) plane of Bi_2_MoO_6_ and the (012) plane of metallic Bi, respectively. Combined with the XRD results and TEM observations, these HRTEM results further support the formation of heterojunction interfaces in the BZ400-2 composite. Furthermore, the EDS analysis (Figure 2g) reveals the presence of Bi, Mo, O, C, and Zn in the BZ400-2 composite, with measured atomic percentages of 6.98%, 0.77%, 9.32%, 78.43%, and 0.57%, respectively. The Bi/Mo atomic ratio is higher than the theoretical stoichiometric ratio of pure Bi_2_MoO_6_, which may be associated with the presence of additional Bi-containing phases and the localized nature of the EDS analysis. The elemental mapping images of BZ400-2 (Figure 2h) further reveal the spatial distribution of Bi, Mo, Zn, C, and O in the analyzed region. Combined with the XRD and TEM results, these observations support the coexistence of the constituent components and their close spatial association in the BZ400-2 composite.

**Figure 3:**
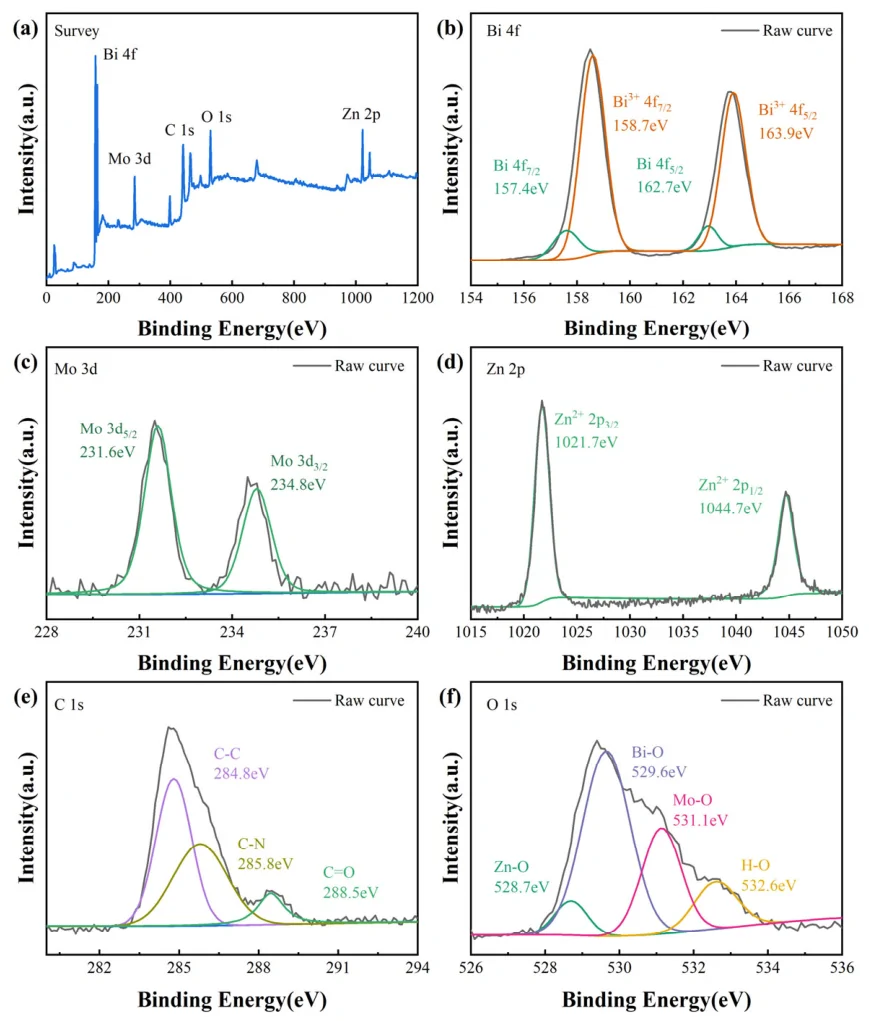
XPS spectra of the prepared BZ400-2: (**a**) survey, (**b**) Bi 4f, (**c**) Mo 3d, (**d**) Zn 2p, (**e**) C 1s, and (**f**) O 1s.

The surface elemental composition and chemical states of the BZ400-2 heterojunction were investigated using X-ray photoelectron spectroscopy (XPS). The binding energies were charge-corrected using the adventitious carbon C 1s peak at 284.8 eV. The full XPS spectrum is shown in Figure 3a, which shows that Bi, Mo, O, Zn and C elements are present in the BZ400-2 heterojunction, which is in good agreement with the analysis results of EDS. As shown in Figure 3b, the peaks at 157.4 and 162.7 eV are assigned to the Bi 4f_7/2_ and Bi 4f_5/2_ components of metallic Bi^0^, respectively [35]. Meanwhile, the peaks at 158.7 and 163.9 eV correspond to the Bi 4f_7/2_ and Bi 4f_5/2_ components of Bi^3+^ species, respectively [36], indicating the coexistence of metallic Bi and Bi^3+^ in the BZ400-2 composite. Based on the fitted Bi 4f peak areas, the relative proportions of Bi^0^ and Bi^3+^ were calculated to be 12.30% and 87.70%, respectively. In the 3d orbital XPS spectrum of Mo (Figure 3c), two peaks located at 231.6 eV and 234.8 eV are attributed to the characteristic binding energies of Mo 3d_5/2_ and Mo 3d_3/2_, respectively, indicating the presence of Mo^6+^ [37]. In the 2p orbital XPS spectrum of Zn (Figure 3d), the two peaks at 1021.7 eV and 1044.7 eV correspond to the binding energies of Zn 2p_3/2_ and Zn 2p_1/2_, respectively, indicating the presence of Zn-O bonds in the BZ400-2 heterojunction. In the 1s XPS spectrum of C (Figure 3e), three peaks at 284.8 eV, 285.8 eV and 288.5 eV are ascribed to C-C bonds, C-N bonds and C=O bonds, respectively, indicating that some organic carbon frameworks were retained during the calcination process [38]. Furthermore, as shown in Figure 3f, the O 1s spectrum can be deconvoluted into four components centered at 528.7, 529.6, 531.1, and 532.6 eV, which may be assigned to Zn-O, Bi-O, Mo-O, and surface hydroxyl or adsorbed oxygen-containing species, respectively [39,40,41].

**Figure 4:**
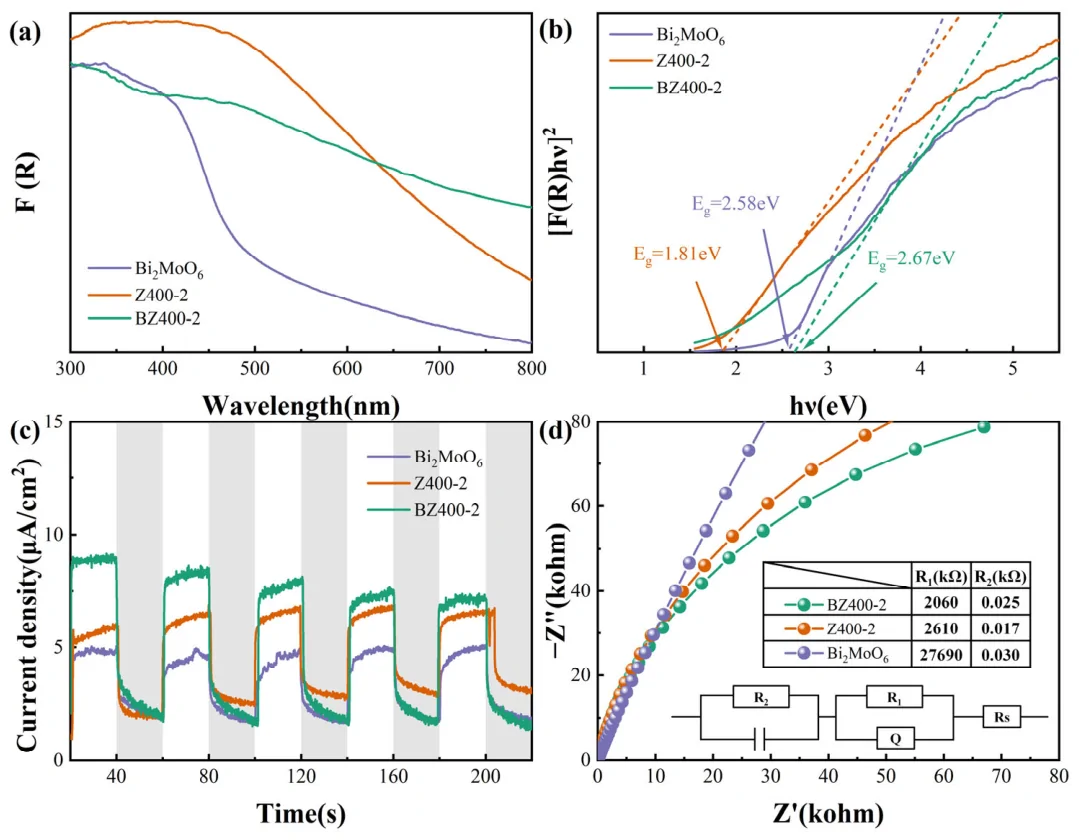
(**a**) UV–vis diffuse reflectance spectra; (**b**) corresponding plots of [F(R)hv]^2^ vs. photon energy; (**c**) time–response current; and (**d**) Nyquist plots of the prepared Bi_2_MoO_6_, Z400-2 and BZ400-2.

The optical properties were analyzed using the UV-Vis diffuse reflectance spectra of Bi_2_MoO_6_, Z400-2, and BZ400-2. As displayed in Figure 4a, the absorption intensity of pure Bi_2_MoO_6_ is at a high level before 400 nm and decreases rapidly between 400 and 500 nm, indicating a poor ability to absorb visible light for degradation of TC. Meanwhile, the Z400-2 exhibits strong light absorption in the 400–500 nm range, which gradually weakens starting around 600 nm; across the entire wavelength range, the absorption intensity of Z400-2 is higher than that of Bi_2_MoO_6_. This suggests that Z400-2 exhibits enhanced light-harvesting capability. For the BZ400-2 heterojunction, the light absorption, especially in the 450–650 nm range, is apparently enhanced. In the wavelength range exceeding 650 nm, BZ400-2 exhibits a higher light absorption intensity than Bi_2_MoO_6_ and Z400-2. We hypothesize that this is due to the surface plasmon resonance (SPR) effect induced by metallic bismuth (Bi); this effect imparts significant light absorption properties to the composite material, thereby enhancing its light-harvesting capability and photo-Fenton-like catalytic activity. Based on the UV-vis DRS results, as described in Figure 4b, the optical band-gap energies (*E_g_*) of the samples were estimated using the Tauc Equation (1) [42].

(*αhν*)*^n^ = A*(*hν* – *E_g_*) (1)

where *α* is the optical absorption coefficient, *h* is Planck’s constant, *v* is the frequency of the incident light, *A* is a proportionality constant, and *E_g_* is the optical band-gap energy. The exponent *n* depends on the nature of the electronic transition. For a direct allowed transition, *n* = 2, whereas for an indirect allowed transition, *n* = 1/2. Accordingly, (*αhν*)^2^ was plotted against *hv*, and the *E_g_* values were obtained by extrapolating the linear portion of the plots to the energy axis. Consequently, the *E_g_* values of Bi_2_MoO_6_, Z400-2, and BZ400-2 were calculated to be 2.58 eV, 1.81 eV, and 2.67 eV, respectively.

To investigate the influence of heterostructure on the photogenerated carrier transfer behavior, the interfacial charge separation and transfer efficiency of the samples were characterized using the transient photocurrent response (I-T) and electrochemical impedance spectroscopy (EIS). Figure 4c shows that the BZ400-2 exhibits the highest current intensity under visible light illumination in comparison with the Z400-2 and pristine Bi_2_MoO_6_, which suggests more efficient separation and transport of photogenerated charge carriers in the heterojunction. It is well known that an equivalent circuit model (illustrated in the inset) is often used to fit the impedance data, where *R_s_* is the solution resistance. In this research system, *R_s_* is the ohmic resistance formed by the electrolyte, substrate, and contact points. *R*_1_ is the charge transfer resistance at the electrode/electrolyte interface. *R*_2_ is the charge migration resistance, equivalent to the resistance encountered by charges transferring from the interior of the material to the surface. Q is the non-ideal capacitance. Generally, the semicircle diameter in the high-frequency region of the Nyquist plot is positively correlated with *R_1_*; the smaller the value, the faster the interfacial charge transfer rate and the higher the separation and injection efficiency of photogenerated carriers [43,44,45]. As shown in Figure 4d, Bi_2_MoO_6_ exhibits the largest semicircle diameter, followed by Z400-2, while BZ400-2 has the smallest semicircle diameter. The equivalent circuit fitting results show that the R_1_ value of BZ400-2 (2060 kΩ) is lower than that of Bi_2_MoO_6_ (27,690 kΩ), confirming that the construction of the heterojunction significantly reduces the charge transfer resistance, which is beneficial to the rapid transport of photogenerated carriers between the interfaces. These EIS results suggest improved interfacial charge transfer behavior in the BZ400-2 composite [46].

**Figure 5:**
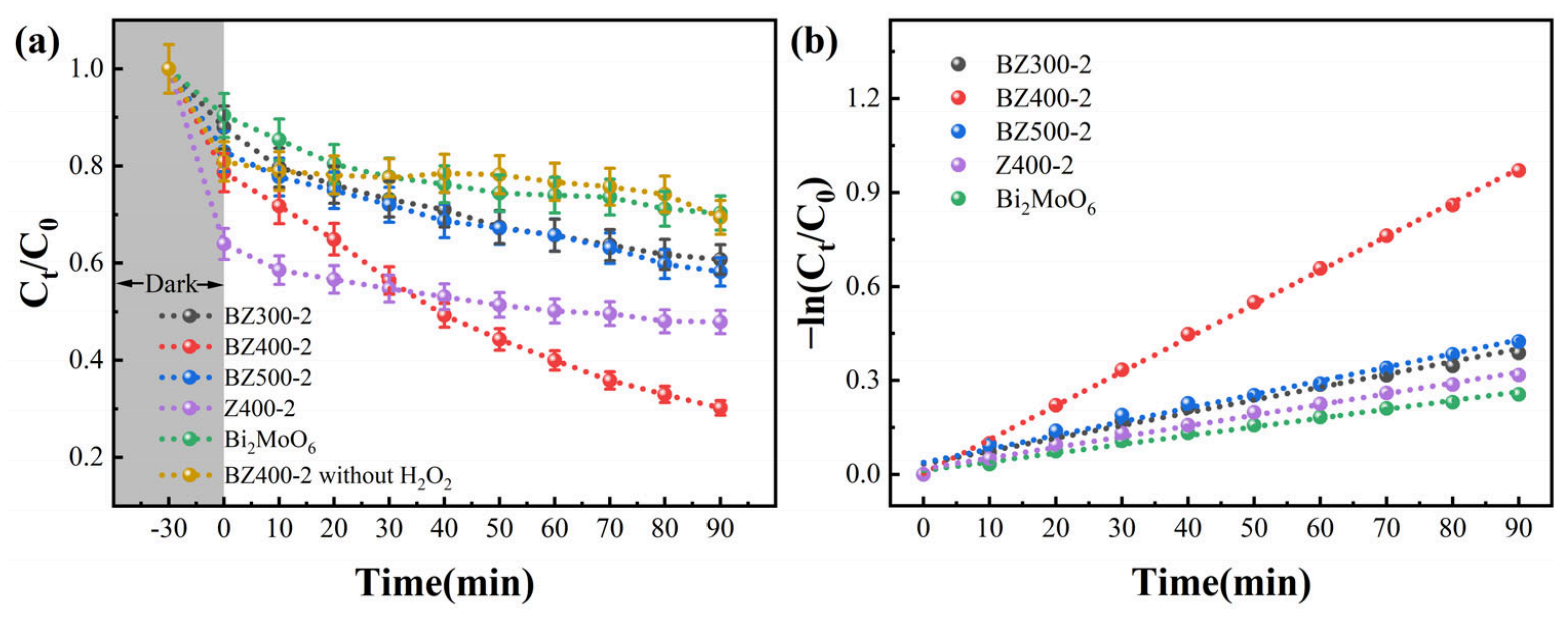
(**a**) Photo-Fenton-like performances of the prepared samples and (**b**) corresponding kinetic fitting curves (catalyst dosage = 0.33 g/L; TC concentration = 50 mg/L).

The photo-Fenton-like performance of the BZ400-2 was evaluated by using tetracycline (TC) as the target pollutant. As shown in Figure S1b, under catalyst-free conditions, it can be seen that the direct photolysis of TC was only 0.5% within 90 min, indicating that structure of TC was highly stable. When H_2_O_2_ was added under visible light irradiation, the TC removal efficiency showed no appreciable change, suggesting that the direct photoactivation of H_2_O_2_ was limited under the experimental conditions. The dark adsorption of the catalyst, as well as the synergistic effect between the catalyst and hydrogen peroxide under dark conditions, were evaluated. In this procedure, BZ400-2 was added to a TC solution and stirred for 30 min; subsequently, hydrogen peroxide was introduced, and the reaction time was reset for another 90 min. It was observed that an adsorption–desorption equilibrium was reached within 15 min, with approximately 19.4% of the TC removed via adsorption. Furthermore, no synergistic effect was found between the BZ400-2 and H_2_O_2_ under dark conditions. As shown in Figure 5a, it was found that the degradation rates of pure Bi_2_MoO_6_ and Z400-2 were 29.65% and 52.12%, respectively. Meanwhile, for the BZ400-2 heterojunction, the degradation performance was enhanced in comparison with the pure Bi_2_MoO_6_ and Z400-2. Among these heterojunctions, the BZ400-2 achieved the highest degradation efficiency, up to 69.8% within 90 min in the photo-Fenton-like system. The degradation process was fitted to a pseudo-first-order kinetic model [47] (formula S2) (Figure 5b); the details regarding the degradation rate constant (k) and *R^2^* values are provided in Table S1. The apparent pseudo-first-order rate constant (*k*) of BZ400-2 was 0.01081 min^−1^, which was approximately 3.86 and 3.16 times that of pristine Bi_2_MoO_6_ (0.00280 min^−1^) and Z400-2 (0.00342 min^−1^), respectively. For comparison, the pure photocatalytic performance of BZ400-2 without H_2_O_2_ is only 30.54% within 90 min under visible light irradiation, indicating that the combination of photocatalysis and Fenton system can efficiently improve the activation of H_2_O_2_ and production of radicals. As shown in Figure 5a, when the carbonization temperature was 300 °C, the degradation rate of TC over the BZ300-2 heterojunction dropped to 40.6% within 90 min; based on prior characterization, it is inferred that insufficient carbonization hindered the formation of the heterojunction. When the carbonization temperature was raised to 500 °C, the degradation rate of TC within 90 min was 41.7%; it is speculated that the higher calcination temperature led to excessive carbonization, thereby reducing the degradation performance. Correspondingly, as displayed in Figure 5b, the reaction kinetic constant of BZ400-2 was 2.65 times and 2.49 times higher than that of BZ300-2 and BZ500-2, respectively. Furthermore, the effects of carbonization times and different doping ratios of Z400-2 on the photo-Fenton-like performance were investigated and are displayed in Figure S1c,d. It is observed that the BZ400-2 achieved the best performance in comparison with the other samples, which suggests that the optimal carbonization time was beneficial for the construction of plasmonic heterojunction. Table 1 compares the TC degradation performance of BZ400-2 with previously reported photocatalytic and photo-Fenton-like systems. Although its degradation efficiency was not the highest, BZ400-2 achieved 69.8% TC removal within 90 min at a relatively low catalyst dosage (0.33 g/L) and a high initial TC concentration (50 mg/L). These results highlight the potential of BZ400-2 for treating relatively high-concentration TC wastewater with reduced catalyst consumption.

**Figure 6:**
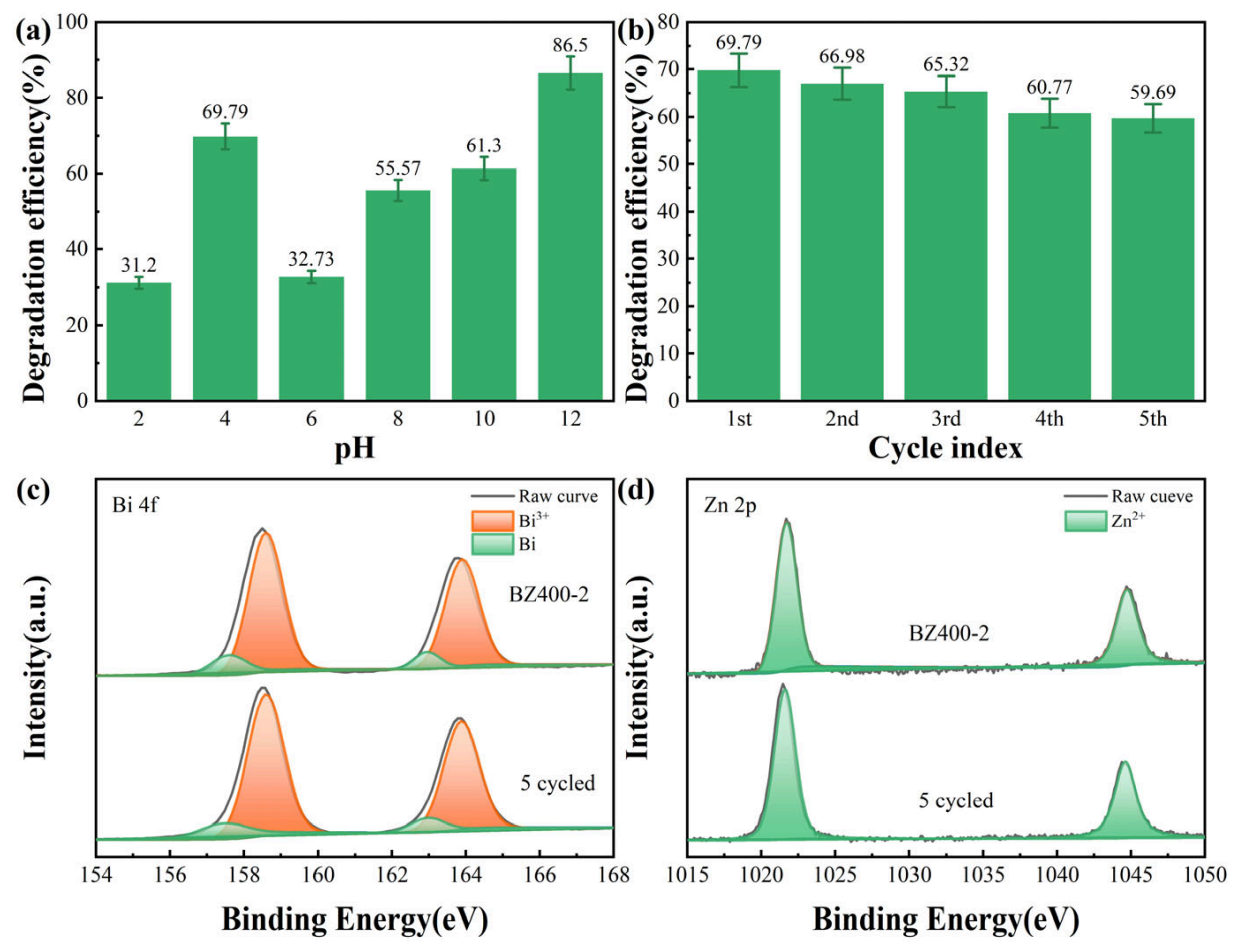
(**a**) Effects of pH values on the photo-Fenton-like performance; (**b**) recycling performance of the BZ400-2 heterojunction. (**c**,**d**) Comparison of high-resolution XPS spectra of (**c**) Bi and (**d**) Zn in pristine BZ400-2 and after 5 cycles.

The effect of initial pH on TC removal efficiency is shown in Figure 6a. As the pH increased from 2 to 4.3, the removal efficiency rose from 31.2% to 69.8%; it dropped to 32.73% when the pH reached 6, and subsequently rose to 86.5% at pH 12. This phenomenon can be attributed to several factors. On the one hand, the pH environment influences the speciation of TC molecules. TC exists primarily as a cation, a zwitterion, and an anion at pH levels below 3.3, between 3.3 and 7.7, and above 7.7, respectively [53]. On the other hand, the pH environment may also affect the activation process of H_2_O_2_. The decrease in the degradation rate observed when shifting the pH from 4 to 2 may be attributed to changes in TC speciation and the erosion of active sites by the strongly acidic environment. At pH 4.3 and 6, TC exists predominantly as a zwitterion; the drop in removal efficiency in this range may be linked to the pH-dependent activation and utilization of H_2_O_2_, which in turn affects the degradation process driven by reactive radicals [53]. Under alkaline conditions, TC deprotonation may facilitate its transformation and enhance its photolysis [54,55], while H_2_O_2_ can further promote TC degradation even without a solid catalyst [56]. Therefore, the higher TC removal observed at pH 12 cannot be attributed solely to BZ400-2. Furthermore, the solution pH influences the interaction between a catalyst and pollutants, as well as the photocatalytic performance [57]. The recycling stability of BZ400-2 heterojunction in photo-Fenton-like system was investigated. As shown in Figure 6b, after five cycles, the degradation efficiency of TC over the BZ400-2 heterojunction was still close to 60%; the loss was 14%. A comparison of the XPS results before and after cycling revealed that the binding energies of the characteristic Bi 4f (Figure 6c) and Zn 2p (Figure 6d) peaks remained unchanged, indicating that the BZ400-2 heterojunction possesses good reusability.

**Figure 7:**
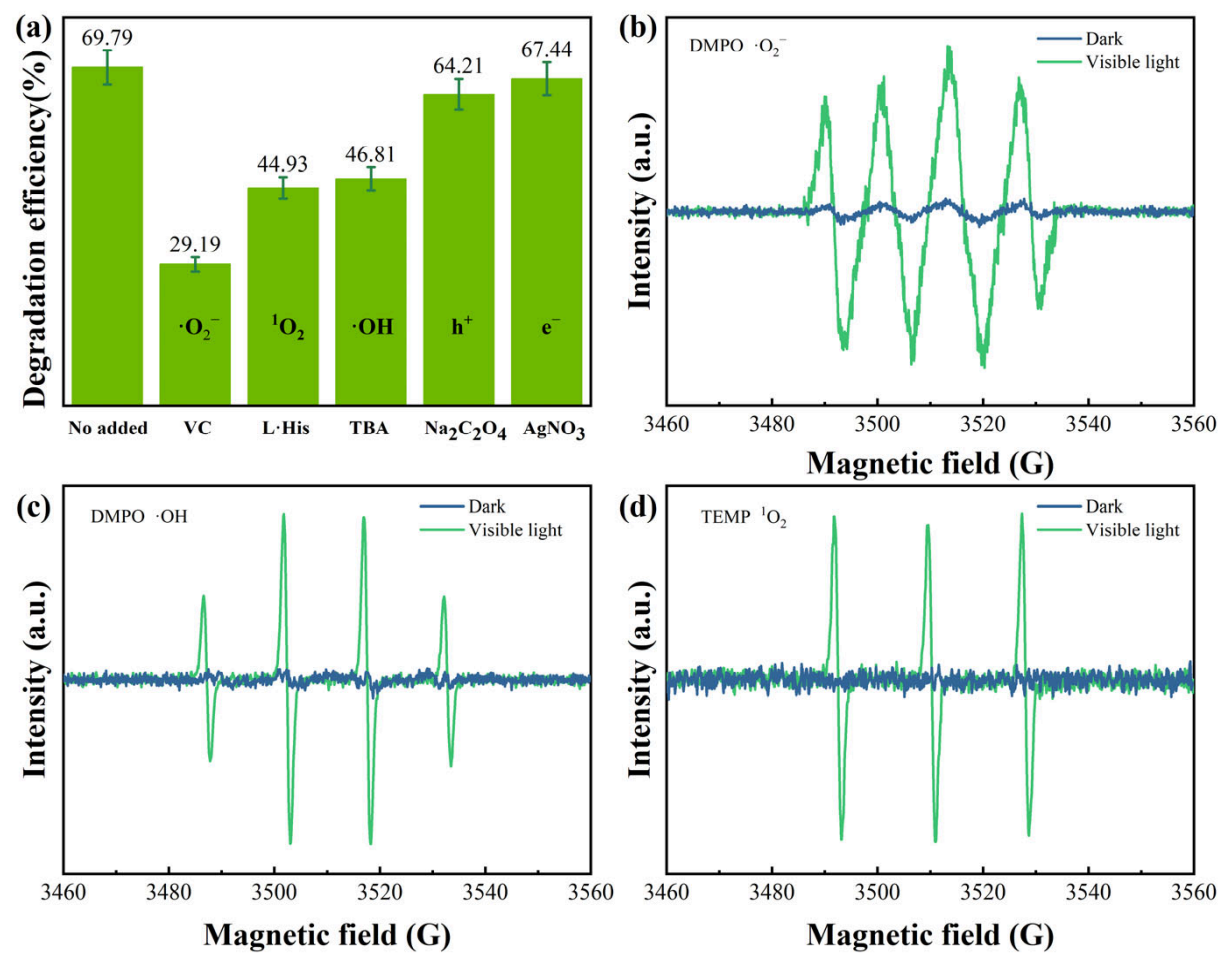
(**a**) Free radical quenching experiment and EPR spectra of (**b**) ·O_2_^−^, (**c**) ·OH, and (**d**) ^1^O_2_.

**Figure 8:**
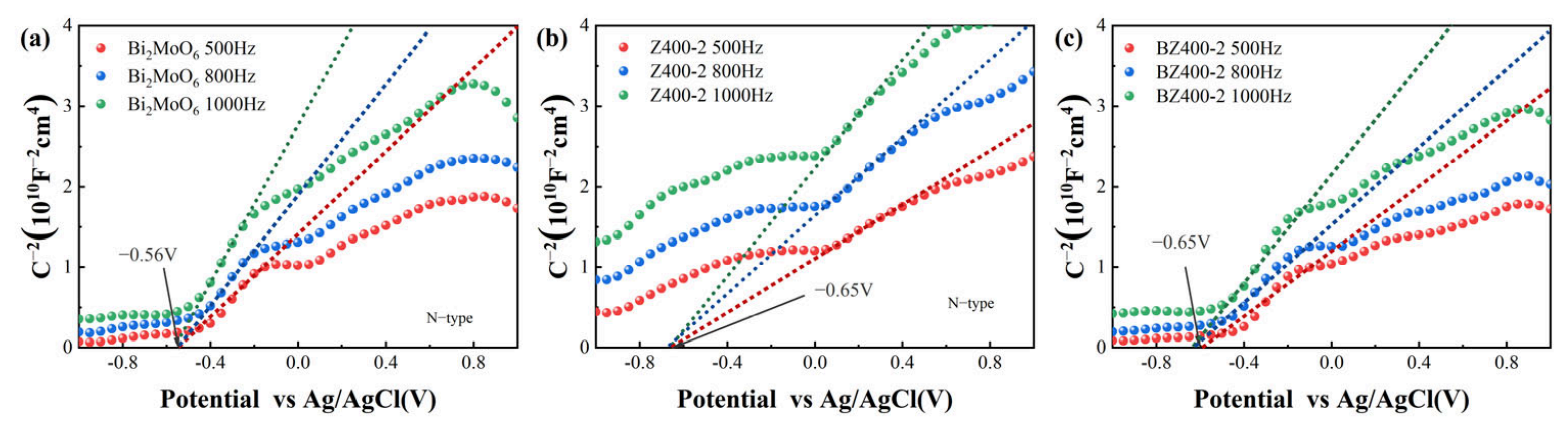
Mott–Schottky curves of the prepared (**a**) Bi_2_MoO_6_, (**b**) Z400-2, and (**c**) BZ400-2 heterojunction.

In order to understand the participation of active substances in the BZ400-2 photo-Fenton-like system, a free radical capture experiment was conducted. Sodium oxalate (Na_2_C_2_O_4_), tert-butanol (TBA), L-histidine (L-His), AgNO_3_ and ascorbic acid (VC) were used as quenchers for holes (h^+^), hydroxyl radicals (·OH), singlet oxygen (^1^O_2_), electrons (e^−^) and superoxide radicals (·O_2_^−^), respectively [58]. As shown in Figure 7a, after the addition of ascorbic acid, L-histidine, and tert-butanol, the TC removal efficiencies decreased to 29.2%, 44.9%, and 46.8%, respectively, while the scavenging of holes and electrons had only slight effects on TC removal. These results suggest that ·O_2_^−^ plays an important role in TC degradation, while ^1^O_2_ and ·OH also participate in the reaction process. The addition of AgNO_3_ only slightly decreased the TC removal efficiency from 69.8% to 67.44%, indicating that electron scavenging had a limited effect on TC removal under the tested conditions. To further verify the generation of ·O_2_^−^, ·OH, and ^1^O_2_ in the BZ400-2 system, ESR spin-trapping measurements were performed using DMPO and TEMP as spin-trapping agents. As shown in Figure 7b, the characteristic signals assigned to ·O_2_^-^ were observed under visible light irradiation, confirming the generation of ·O_2_^−^. In Figure 7c, a characteristic quartet with an intensity ratio of approximately 1:2:2:1 is shown, corresponding to ·OH and confirming the formation of ·OH. In addition, Figure 7d shows a characteristic 1:1:1 triplet assigned to the ^1^O_2_ under visible light irradiation, demonstrating the generation of ^1^O_2_. These ESR results are consistent with the radical quenching experiments and further confirm the participation of ·O_2_^−^, ·OH, and ^1^O_2_ in the reaction system. The Mott–Schottky plots of Bi_2_MoO_6_, Z400-2 and BZ400-2 recorded at 500, 800, and 1000 Hz are shown in Figure 8a–c. The positive slopes observed for Bi_2_MoO_6_ and Z400-2 are consistent with their n-type semiconductor characteristics [59]. Frequency dispersion is observed in the Mott–Schottky plots, where the capacitance response and extrapolated intercepts vary with the frequency due to interfacial inhomogeneity. Based on the extrapolation of the approximately linear regions of the plots, the apparent effective flat-band potentials (Efb) for Bi_2_MoO_6_, Z400-2, and BZ400-2 were estimated to be approximately −0.56, −0.65, and −0.65 V (vs. Ag/AgCl), respectively. The E_fb(SHE)_ values for Bi_2_MoO_6_, Z400-2, and BZ400-2 were calculated using formula (2).

*E_fb_*_(*SHE*)_ = *E_fb_*_(*Ag/AgCl*)_ + 0.197 (2)

Generally, the *E_CB_* of n-type semiconductor is about 0.1–0.3 V less than the *E_fb_* [60,61]. In this study, an offset of 0.20 V was adopted. Therefore, the *E_CB_* of Bi_2_MoO_6_, Z400-2 and BZ400-2 were calculated to be −0.56 V, −0.65 V and −0.65 V (vs. standard hydrogen electrode, SHE). Correspondingly, the *E_VB_* of Bi_2_MoO_6_, Z400-2 and BZ400-2 were calculated to be 2.02 V, 1.16 V and 2.02 V, respectively. Notably, the value of 1.16 V for Z400-2 represents an apparent estimate based on the optical response of the ZnO@C composite rather than the intrinsic valence band potential of ZnO.

**Figure 9:**
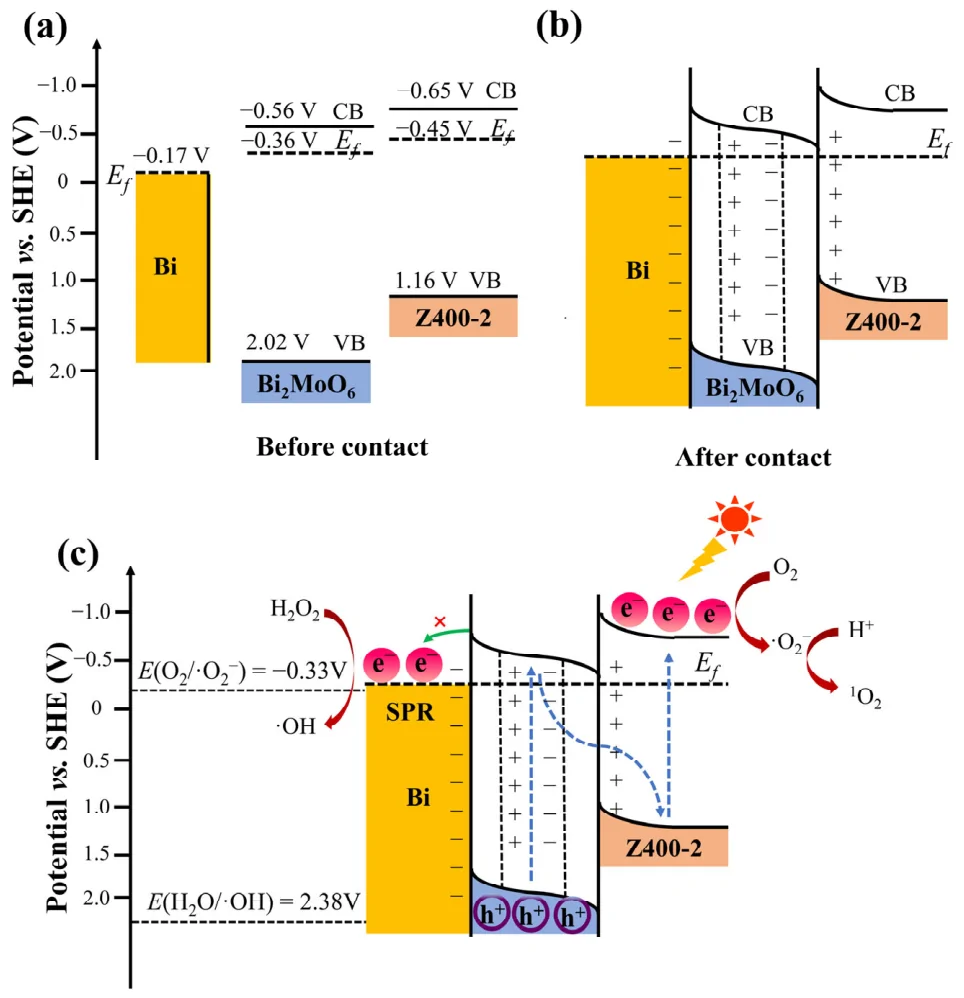
Band alignments of Bi, Bi_2_MoO_6_, and Z400-2 before (**a**) and after (**b**) formation of heterojunction; (**c**) schematic diagram of photo-Fenton-like mechanism over the BZ400-2 heterojunction.

Based on the above results, the photo-Fenton-like mechanism over the BZ400-2 is proposed and displayed in Figure 9. The proposed band alignment of Bi, Bi_2_MoO_6_ and ZnO@C before interfacial contact is illustrated in Figure 9a. The Fermi-level potential of metallic Bi was adopted as approximately −0.17 V vs. NHE based on previously reported literature [62,63]. Upon the formation of heterojunctions, charge transfer occurs across the interfaces to equilibrate the Fermi levels, driven by the migration of intrinsic charge carriers, resulting in band bending among Bi, Bi_2_MoO_6_, and ZnO@C. As shown in Figure 9b, the energy bands of ZnO@C bend upward near the interface with Bi_2_MoO_6_; simultaneously, the conduction band of Bi_2_MoO_6_ bends downward near the interface with ZnO@C but upward near the interface with Bi, creating a Schottky barrier that impedes the migration of charge carriers toward the Bi. Based on the band structure analysis and ROS identification results, a possible S-scheme-like interfacial charge transfer pathway is proposed for BZ400-2. Under visible light irradiation, photogenerated electrons in Bi_2_MoO_6_ may recombine with holes in ZnO@C across the heterointerface, while the remaining electrons and holes retain sufficient redox ability to participate in ROS generation. Accordingly, photogenerated holes with relatively strong oxidation ability may remain on Bi_2_MoO_6_, while photogenerated electrons with sufficient reduction ability may accumulate on ZnO@C. Since the conduction band potential of ZnO@C is approximately −0.65 V vs. SHE, which is more negative than the electrode potential of *E*(O_2_/·O_2_^−^) (−0.33 V vs. SHE), the photoinduced electrons can thermodynamically generate ·O_2_^−^, then partially form ^1^O_2_. However, the valence band potential of Bi_2_MoO_6_ (2.02 V vs. SHE) is less positive than the oxidation potential of H_2_O/·OH (2.38 V vs. SHE at pH 7) [64], suggesting that photogenerated holes cannot readily oxidize H_2_O to generate ·OH. At the interface, the contact between metallic Bi^0^ and semiconducting Bi_2_MoO_6_ induces a redistribution of interfacial charges. Furthermore, under visible light irradiation, the plasmon excitation occurring on the surface of metallic bismuth generates high-energy electrons; these electrons are then transferred to the adsorbed H_2_O_2_, thereby activating it. This activated H_2_O_2_ significantly promotes the generation of reactive oxygen species (ROS), with radicals such as ·OH subsequently detected via EPR and quenching experiments. Meanwhile, the carbon component may provide an additional pathway for interfacial charge transfer during the photocatalytic H_2_O_2_ activation process. Ultimately, TC is attacked by reactive oxygen species and gradually decomposed into smaller organic intermediates.

**Figure 10:**
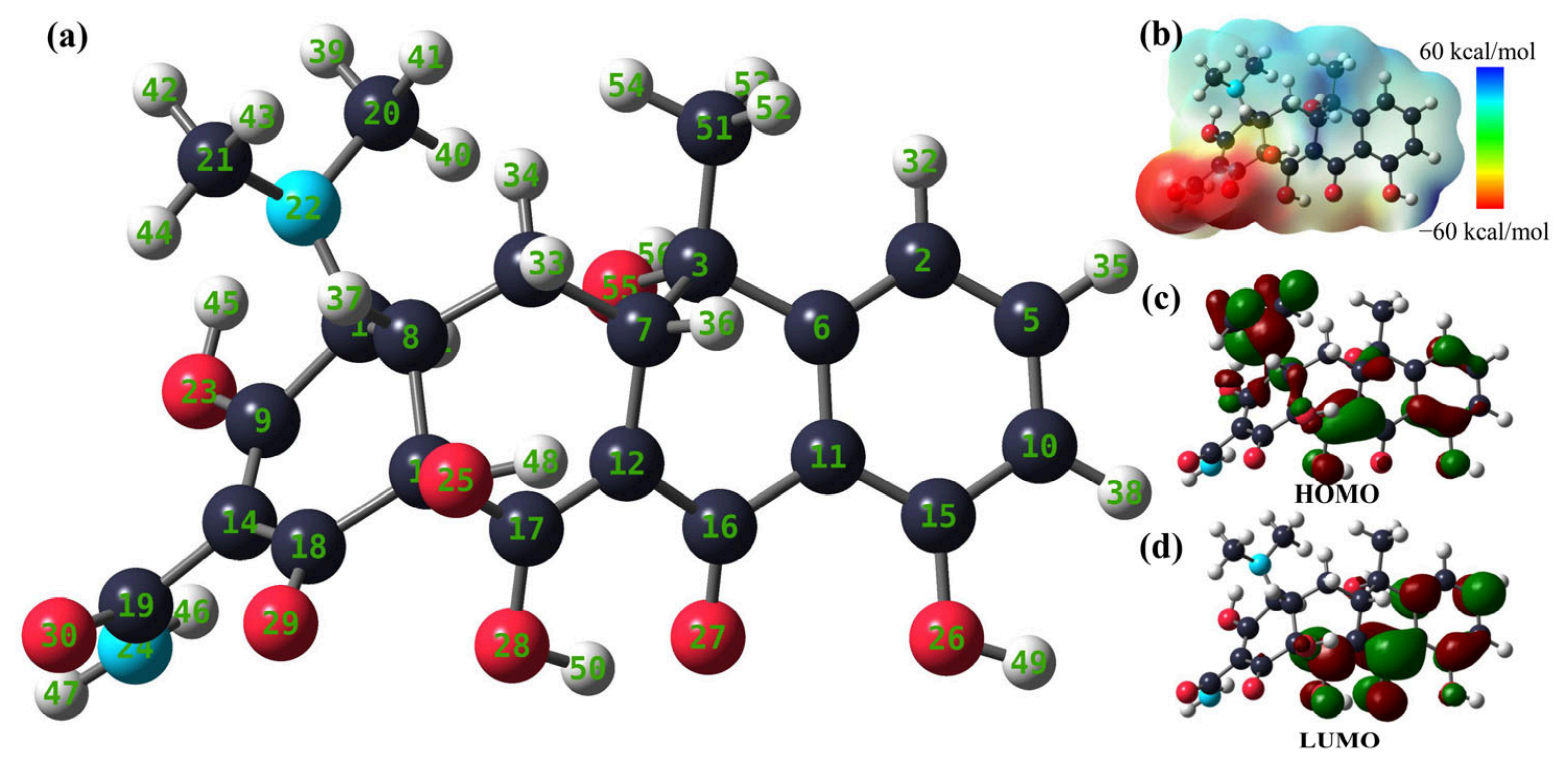
(**a**) Optimized chemical structure, (**b**) electrostatic potential, and (**c**) HOMO and (**d**) LUMO orbitals of TC.

To further demonstrate the degradation process of TC, systematic quantum chemical calculations were performed based on density functional theory (DFT), including a molecular structure optimization (Figure 10a), surface analysis of molecular electrostatic potential (ESP), frontier orbital energy level (HOMO/LUMO) assessment, and Fukui function calculations. As shown in Figure 10b, the electrostatic potential (ESP) analysis reveals that the regions corresponding to the aromatic ring (D-ring), the methyl and hydroxyl groups on the C-ring, and the dimethylamino group on the A-ring exhibit a positive electrostatic potential (indicated in blue). This suggests that these regions are relatively electron-deficient, capable of attracting negative charges, and generally more susceptible to nucleophilic attack. In contrast, the regions corresponding to the diketone structure—spanning the C- and B-rings—and the carboxamide group on the A-ring exhibit a negative electrostatic potential (indicated in red). This indicates that these regions are relatively electron-rich, repelling negative charges while showing a stronger attraction toward positive charges. According to frontier molecular orbital theory, the highest occupied molecular orbital (HOMO) reflects a molecule’s ability to donate electrons, whereas the lowest unoccupied molecular orbital (LUMO) reflects its ability to accept electrons. As shown in Figure 10c,d, the HOMO orbitals of TC are mainly delocalized in the diketone conjugated structure of rings B and C and the dimethylamino group on the A-ring, making them susceptible to attack by electrophilic free radicals; the LUMO orbitals are mainly localized around the phenolic hydroxyl group on the D-ring and the methyl and hydroxyl groups on the C-ring, making them more susceptible to attack by nucleophilic free radicals. Furthermore, the calculated Fukui function values of the atoms in TC are listed in Table S2. Among them, O27 exhibits the highest radical attack index (f^0^ = 0.0778), followed by C16 (0.0725), O28 (0.0680), and C17 (0.0635), suggesting that these sites are relatively susceptible to radical attack.

**Figure 11:**
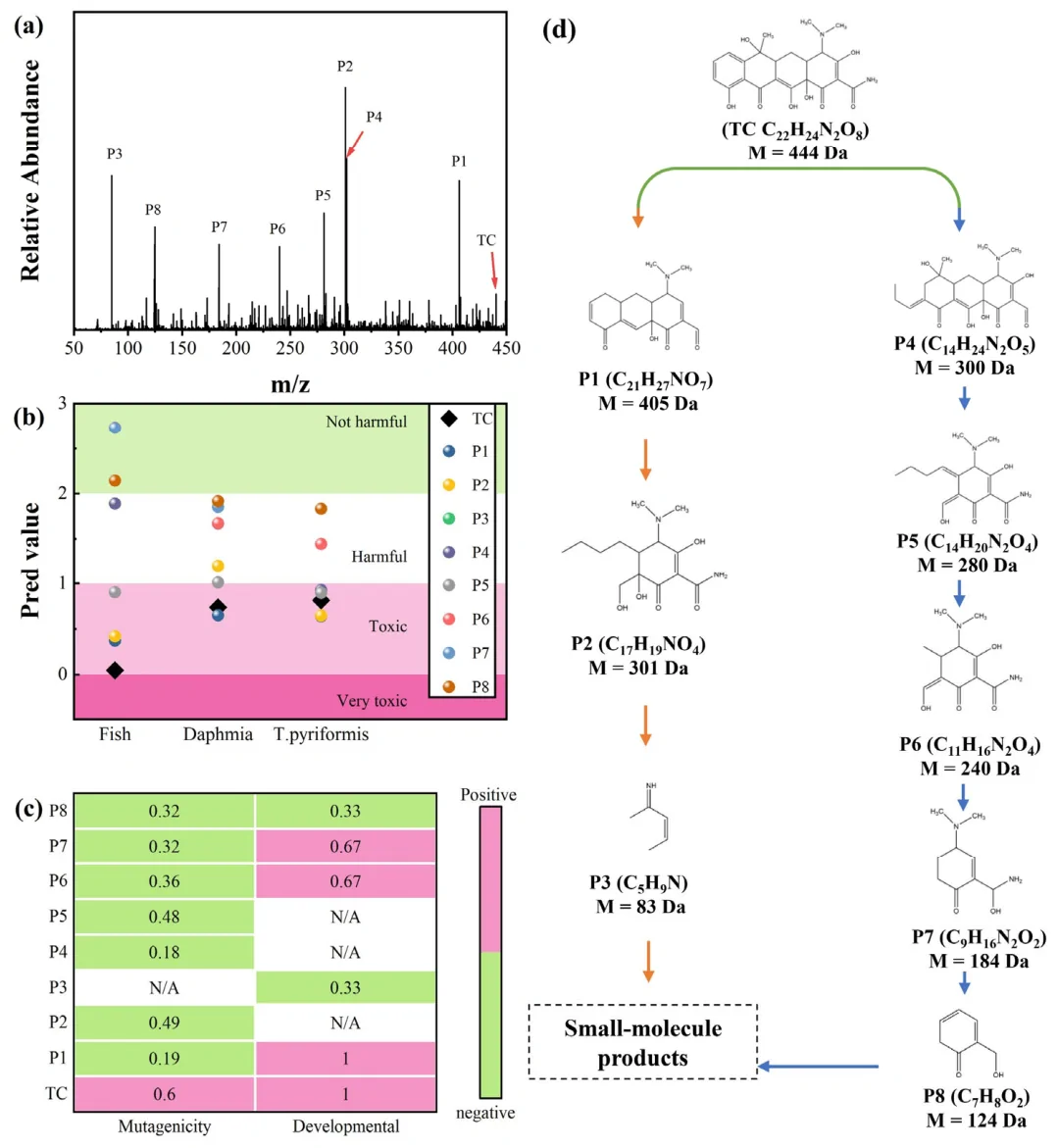
(**a**) High-resolution mass spectra of TC degradation products; (**b**) the acute toxicity (Log_LC50_ of fathead minnow (96 h), Log_LC50_ of Daphnia magna (48 h), Log_IGC50_ of Tetrahymena pyriformis (48 h)); (**c**) developmental toxicity and mutagenicity; (**d**) degradation pathway of TC.

Additionally, high-resolution mass spectrometry (HRMS) was employed to investigate the degradation intermediates of TC (Figure 11a). In positive-ion electrospray ionization mode, the degradation intermediates were detected as [M+H]^+^ ions; therefore, the corresponding neutral molecular mass could be calculated by subtracting the mass of a proton (approximately 1.0073 Da) from the measured mass-to-charge (*m*/*z*) value [65]. Specifically, TC was detected as the protonated ion [M+H]^+^ at *m*/*z* 445, corresponding to a nominal neutral molecular mass of 444 Da. Accordingly, the nominal neutral molecular masses of TC and the proposed intermediates are reported as 444, 405, 301, 300, 280, 240, 184, 124, and 83 Da, respectively, in the proposed degradation pathway. According to previous studies [66,67], the degradation process of TC mainly involves dehydration, hydroxylation, demethylation, decarbonylation and oxidation. Combined with the DFT calculation results, the C=C bond, phenolic hydroxyl group and amino functional group have a high electron density and are the main targets of active free radicals [68,69]. Based on the comprehensive active site analysis, two possible TC degradation pathways are proposed and displayed in Figure 11d. In pathway I, TC first undergoes deamination and other reactions under the action of free radicals to generate P1. P1 is further transformed into P2 through ring opening and bond breaking. Subsequently, under the continuous action of ROS, P2 undergoes further transformations to generate the smaller intermediate P3. In pathway II, TC undergoes dehydration and ring opening to generate P4; P4 is further converted into P5 under the action of free radicals, and then reacts sequentially to transform into P6 and P7. Subsequently, under the action of reactive species, P7 is transformed into P8, which may undergo further degradation to form smaller molecular products.

The acute toxicity, developmental toxicity and mutagenicity of TC and degradation intermediates were predicted by using T.E.S.T (5.1.2.0) toxicity assessment software based on the quantitative structure–activity relationship (QSAR) method. Among these, the comparative indicators for acute toxicity are the 96 h Log_LC50_ for the fathead minnow, the 48 h Log_LC50_ for Daphnia magna, and the 48 h Log_IGC50_ for Tetrahymena pyriformis. According to the toxicity classification criteria in Table S4 [70], TC exhibited predicted acute toxicity toward the fathead minnow, *Daphnia magna*, and T. pyriformis, with log_LC50_ values of 0.04576 and 0.73560 for the first two organisms and a log_IGC50_ value of 0.81291 for T. pyriformis, respectively (Figure 11b). The predicted LC_50_/IGC_50_ values of most degradation intermediates were higher than those of TC, suggesting an overall reduction in acute toxicity, with only a few exceptions observed for P1 (Daphnia magna and T. pyriformis) and P2 (T. pyriformis). Regarding developmental toxicity (Figure 11c), TC and P1 showed positive predictions (1.00), while P6 and P7 also exceeded the 0.5 threshold (0.67). In contrast, P3 and P8 showed negative predictions (0.33), whereas no predictions were available for P2, P4, and P5. The mutagenicity predictions for P1, P2, and P4-P8 were below the threshold of 0.5, suggesting a reduced mutagenic potential compared with TC. No prediction was available for P3. Therefore, the BZ400-2 heterojunction exhibits effective TC degradation performance, while the QSAR predictions suggest reduced acute toxicity for most of the identified degradation intermediates.

## 4. Conclusions

In summary, a novel ZnO@C/Bi/Bi_2_MoO_6_ heterojunction was successfully prepared by using ZIF-8 as a precursor through a carbonization and hydrothermal route. The formation of a heterojunction structure effectively enhanced the separation efficiency of photogenerated charge carriers, and the capacity for visible light absorption was also notably improved. As a result, the BZ400-2 achieved the best photo-Fenton-like performance, reaching 69.8% TC degradation within 90 min under visible light irradiation. The apparent pseudo-first-order rate constant of BZ400-2 reached 0.01081 min^−1^, approximately 3.86 and 3.16 times that of pristine Bi_2_MoO_6_ and Z400-2, respectively. The radical quenching experiments and ESR measurements suggest that ·O_2_^−^ plays an important role in TC degradation, while ^1^O_2_ and ·OH also participate in the reaction process. Furthermore, the degradation pathway of TC was analyzed and proposed based on DFT calculations and HRMS. Meanwhile, QSAR-based toxicity predictions suggest that most of the identified degradation intermediates exhibit lower acute toxicity than TC. This study provides a new strategy for constructing heterogeneous photo-assisted Fenton-like systems by using MOFs as precursors to develop efficient advanced oxidation processes.

## Figures and Tables

**Table 1:** Comparison of the TC degradation performances by the previously reported heterojunction in PC/PF system.

Photocatalysts	Catalyst Dosage (g/L)	Initial TC Concentration (mg/L)	Light	Reaction System	Photocatalytic Performance	Ref.
ZnO nanorod/ Bi_2_MoO_6_/ MIL-101(Fe)	0.38	20	20 W, LED	PC	60 min, 77.7%	[48]
ZnO/Bi_2_MoO_6_/ZIF-67	0.40	40			90 min, 90.3%	[49]
Bi_2_MoO_6_/ZnSnO_3_/ZnO	1.67	50	125 W, UV light	PF	120 min, ~80%	[50]
Fe_2_O_3_@C/Bi_2_MoO_6_	0.80	25	500 W, Xe lamp		93.2%, 50 min	[26]
ZnO/Ag/Bi_2_MoO_6_	1.00	20	300 W, Xe lamp	PC	120 min, 97.3%	[51]
ZnO QDs/Bi_2_MoO_6_	0.2	20			120 min, 94.20%	[52]
Ce-doped Bi_2_MoO_6_	0.63	40		PF	90.7%, 40 min	[12]
ZnO@C/Bi/Bi_2_MoO_6_	0.33	50			90 min, 69.8%	This work

## Data Availability

The raw data supporting the conclusions of this article will be made available by the authors on request.

## Supplementary Materials

The following supporting information can be downloaded at: https://www.mdpi.com/article/10.3390/nano16211393/s1, Text S1 Experimental: S1.1 Characterizations, S1.2 Tetracycline hydrochloride test method, S1.3 Kinetic model, S1.4 Other tests, S1.5 Computational method; Figure S1: (a) XRD patterns of obtained ZIF-8 and ZnO@C with different calcination temperatures, (b) Degradation of tetracycline by dark adsorption, pure light and H_2_O_2_ only, (c) Photo-Fenton-like performance of these samples with different calcination times and (d) different doping ratio; Table S1 k and R2 of degradation kinetic curves of TC over these different samples; Text S2 TC degradation products and their toxicity analysis; Table S2 Fukui indices of TC-HCl; Table S3 Toxicity prediction of tetracycline hydrochloride degradation intermediates; Table S4 Acute toxicity prediction classification criteria. References [71,72,73,74,75,76,77] are cited in the supplementary materials.
