# Supplementary material for: Interface-Engineered ZIF-8-Derived ZnO@C/Bi/Bi2MoO6 Heterojunctions for Boosting Photo-Fenton-like Performance
Source: Nanomaterials (Basel). 2026 Oct 29;16(21):1393. doi: 10.3390/nano16211393 (PMC13765004; doi:10.3390/nano16211393)
Supplement: Supplementary file 1 [file nanomaterials-16-01393-s001.zip › nanomaterials-4609845-supplementary.pdf]

Supplementary Materials

Interface-Engineered ZIF-8-Derived ZnO@C/Bi/Bi_2MoO_6 Heterojunctions for Boosting Photo-Fenton-Like Performance

Yixuan Hu [1], Jun Ke [1,2,*], Zhaoyang Yan [1] and Jie Liu [1]

1 Department of Environmental Science and Engineering, North China Electric Power University, Baoding 071003, China
2 MOE Key Laboratory of Resources and Environmental Systems Optimization, North China Electric Power University, Beijing 102206, China
* Correspondence: jke@ncepu.edu.cn

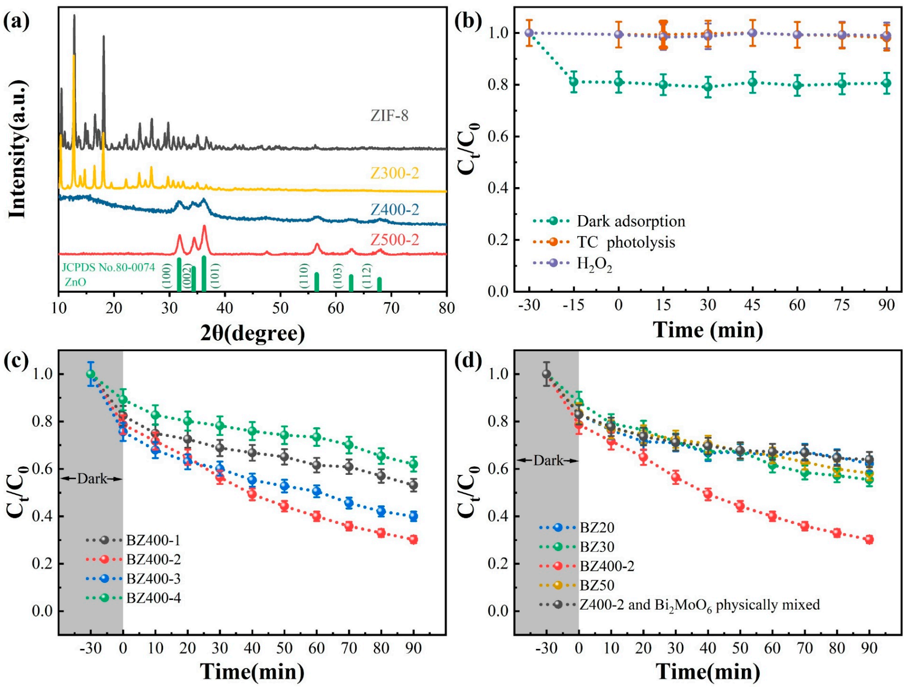

Figure S1. (a) XRD patterns of obtained ZIF-8 and ZnO@C with different calcination temperatures, (b) Degradation of tetracycline by dark adsorption, pure light and H_2O_2 only. (c) Photo-Fenton-like performance of these samples with different calcination times and (d) different doping ratio.

The effect of calcination time on degradation is shown in **Fig. S1c**. Taking 400 degrees Celsius as an example, the degradation efficiency first increases and then decreases with increasing calcination time. ZIF-8 showed the best effect after 2 hours of calcination. Therefore, the subsequent calcination time was selected as 2 hours. **Fig. S1d** compares the TC degradation performance of composite materials with varying doping levels in a photo-Fenton system. As the doping proportion of Z400-2 in the composites increased, the TC degradation rate initially rose and then declined. Notably, the BZ400-2 composite (with a 40% doping level) exhibited the best photo-Fenton degradation performance, achieving a degradation rate of 69.8% after 90 minutes of reaction; consequently, a 40% doping proportion was selected for subsequent experiments. Analysis indicates that at low ZIF-8 doping levels, a scarcity of active sites resulted in poor degradation performance, whereas excessive ZIF-8 doping masked active sites, thereby further reducing the degradation rate. Additionally, the physical mixture control was prepared by mixing Z400-2 and Bi_2MoO_6 at a mass ratio of 40:60 (w/w). The mixture exhibited a TC removal

efficiency of only 36% after 90 min in the photo-Fenton system, indicating that simple physical mixing could not reproduce the enhanced catalytic performance of the BZ400-2 heterojunction.

Table S1. *k* and R^2 of degradation kinetic curves of TC over these different samples.

Sample	k(10^{-3}min^{-1})	R^2	Sample	k(10^{-3}min^{-1})	R^2
Bi_2MoO_6	2.80	0.9912	BZ400-2	10.81	0.9995
Z400-2	3.42	0.9924	BZ500-2	4.35	0.9837
BZ300-2	4.08	0.9814	--	--	--

Text S1 Experimental

S1.1 Characterizations

The phase composition of the as-prepared samples was determined by X-ray diffraction (XRD, Bruker D8 Advance, Germany). The surface morphology and elemental distribution were examined using field-emission scanning electron microscopy (FE-SEM, ZEISS Gemini SEM 300, Germany), while the microstructure and lattice fringes were further characterized by transmission electron microscopy (TEM) and high-resolution TEM (HRTEM, JEM-F200, Japan). UV-Vis-NIR diffuse reflectance spectra were recorded on a UV-visible spectrophotometer (Shimadzu UV-3600i Plus, Japan) using $BaSO_4$ as the reference over the wavelength range of 200–800 nm, aiming to evaluate the optical absorption properties. The surface chemical composition and elemental valence states were analyzed by X-ray photoelectron spectroscopy (XPS, Thermo Fisher Scientific K-Alpha, USA). The concentration of tetracycline was determined via high-performance liquid chromatography (HPLC, Thermo Dionex Ultimate 3000, USA), and the resulting degradation intermediates were identified by high-resolution mass spectrometry (HRMS, Thermo Scientific Q Exactive Orbitrap, USA) to elucidate the possible degradation pathway. Electrochemical measurements, including electrochemical impedance spectroscopy (EIS), Mott–Schottky analysis, and transient photocurrent response (i-t), were performed on an electrochemical workstation (CHI660E, Chenhua, China) using a standard three-electrode system. Electron paramagnetic resonance (EPR) was performed using a paramagnetic resonance spectrometer (Bruker EMXplus-6/1, Germany).

S1.2 Tetracycline hydrochloride test method

The concentration of tetracycline (TC) was determined via high-performance liquid chromatography (HPLC) equipped with a C18 reversed-phase column (150 mm × 4.6 mm, 5 μm). The mobile phase consisted of 0.1% formic acid aqueous solution (A) and methanol (C). The gradient elution program was as follows: 0–1.5 min, 95% A; 2.0 min, 35% A; 7.5 min, 65% A; and 8.0-10.0 min, 95% A. The calibration range was set at 2-50 mg/L TC. The chromatographic separation was carried out at a flow rate of 1.0 mL/min, with the column temperature maintained at 35 ℃ and an injection volume of 10 μL. The eluted analyte was monitored using a UV detector at a fixed wavelength of 355 nm. The retention time of TC was approximately 4.90 min. The limit of detection (LOD) and limit of quantification (LOQ) were 2 and 8 ng/L, respectively.

The residual TC concentration (C, mg/L) during the degradation process was determined by interpolating the chromatographic peak signal into the standard calibration curve, which was established as y = 0.3435x - 1.0894 (R^2 = 0.9999), where y represents the peak area and x denotes the corresponding TC concentration. The degradation efficiency of TC was then calculated according to Eq. (S1):

$$\text{Degradation efficiency} = (C0 - Ct)\ /C0 \times 100\% \qquad \text{(S1)}$$

In the formula, C_0 is the initial concentration of tetracycline, and C_t is the concentration of tetracycline at time t.

S1.3 Kinetic model

The first-order kinetic model (Equation S2) used to evaluate the degradation effect is as follows:

$$-ln\frac{C_t}{C_0} = k_1 t \tag{S2}$$

Where *t* refers to the illumination time, C_0 refers to the initial concentration, C_t represents the sample concentration at time t, and k_1 represent the reaction rate constants of the first-order kinetic model.

S1.4 Other tests

To investigate the effect of initial pH on the degradation performance, the solution pH was adjusted to the desired value using 0.1 mol/L HCl or 0.1 mol/L NaOH prior to illumination. For the radical quenching experiments, various scavengers were introduced into the reaction system to identify the predominant reactive species, including sodium oxalate ($Na_2C_2O_4$, 4.98 mM) for h^+, tert-butanol (TBA, 348 mM) for ·OH, L-histidine (L-His, 4.30 mM) for $^1O_2$, $AgNO_3$ (3.92 mM) for e^-, and ascorbic acid (VC, 3.79 mM) for $\cdot O_2^-$. After each degradation run, the used catalyst was collected by filtration and washed alternately with deionized water and anhydrous ethanol for three times, followed by drying in an oven prior to the next cycle. The reusability of the catalyst was evaluated over five consecutive cycles, and each cycle was conducted in triplicate to ensure reproducibility. ESR measurements were performed in aqueous solution using DMPO (100 mM) as the spin-trapping agent for both $\cdot O_2^-$ and ·OH, while TEMP (100 mM) was used for $^1O_2$ detection. The experiments were conducted with 10 mg of BZ400-2 and 10 μL of H_2O_2 (30 wt%) under visible-light irradiation (300 W xenon lamp, $\lambda > 420$ nm) for 10 min before ESR analysis.

S1.5 Computational method

All density functional theory (DFT) calculations were performed using the Gaussian 16 software package[1]. The B3LYP hybrid functional [2] was adopted, incorporating the D3BJ empirical dispersion correction [3] to account for non-covalent interactions. Geometry optimizations and subsequent vibrational frequency analyses were carried out at the def2SVP level [4] for all atoms, while single-point energy calculations were refined at the B3LYP-D3BJ/def2TZVP level of theory. To better mimic the aqueous solvation environment, the SMD implicit solvation model [5] was applied throughout both the optimization and frequency calculation stages, with water selected as the solvent. Additionally, Fukui function analyses [6] and electrostatic potential (ESP) mapping were conducted using the Multiwfn [7] and VMD [8] programs, respectively. The Fukui indices were calculated using the neutral molecular form of TC as a simplified model. Considering the pH-dependent speciation of TC under the experimental conditions (pH 4.3), these results were used to qualitatively identify potential reactive sites.

Text.S2 TC degradation products and their toxicity analysis

Table S2. Fukui indices of TC-HCl.

Atom	q(N)	q(N+1)	q(N-1)	f^-	f^+	f^0	CDD
1(C)	0.0244	0.0229	0.0274	0.003	0.0014	0.0022	-0.0016
2(C)	-0.0548	-0.0837	0.0224	0.0772	0.0289	0.0531	-0.0482
3(C)	0.0922	0.0866	0.1001	0.0079	0.0056	0.0068	-0.0023
4(C)	-0.0421	-0.0469	-0.037	0.0051	0.0048	0.0049	-0.0003
5(C)	-0.0245	-0.0912	0.021	0.0455	0.0668	0.0561	0.0213
6(C)	0.0071	-0.0325	0.0301	0.023	0.0395	0.0313	0.0165
7(C)	-0.0133	-0.0197	-0.0023	0.011	0.0064	0.0087	-0.0046
8(C)	-0.0229	-0.0258	-0.0198	0.0032	0.0028	0.003	-0.0003
9(C)	0.1258	0.1226	0.1285	0.0027	0.0032	0.0029	0.0005
10(C)	-0.0581	-0.093	-0.011	0.0471	0.0348	0.041	-0.0122
11(C)	-0.0409	-0.0731	0.0124	0.0534	0.0322	0.0428	-0.0212
12(C)	-0.0527	-0.0828	0.0189	0.0716	0.03	0.0508	-0.0415
13(C)	0.0659	0.0552	0.0708	0.0048	0.0108	0.0078	0.0059
14(C)	-0.0722	-0.0758	-0.0681	0.0042	0.0036	0.0039	-0.0006
15(C)	0.0884	0.0437	0.1538	0.0654	0.0447	0.0551	-0.0207
16(C)	0.1278	0.0038	0.1489	0.0211	0.124	0.0725	0.1029
17(C)	0.1017	0.0239	0.1509	0.0492	0.0778	0.0635	0.0286
18(C)	0.1237	0.1188	0.1269	0.0032	0.0049	0.0041	0.0017
19(C)	0.1514	0.1494	0.1532	0.0018	0.002	0.0019	0.0001
20(C)	-0.0358	-0.0373	-0.0307	0.0052	0.0014	0.0033	-0.0037
21(C)	-0.0337	-0.0349	-0.0293	0.0044	0.0012	0.0028	-0.0032
22(N)	-0.0496	-0.0509	-0.0348	0.0148	0.0013	0.0081	-0.0134
23(O)	-0.1692	-0.1739	-0.163	0.0062	0.0047	0.0055	-0.0015
24(N)	-0.1251	-0.1265	-0.1236	0.0015	0.0014	0.0015	-0.0001
25(O)	-0.2418	-0.2653	-0.2251	0.0167	0.0235	0.0201	0.0068
26(O)	-0.1893	-0.2195	-0.1079	0.0813	0.0303	0.0558	-0.0511
27(O)	-0.2714	-0.3946	-0.239	0.0325	0.1232	0.0778	0.0907
28(O)	-0.197	-0.2637	-0.1277	0.0693	0.0667	0.068	-0.0027
29(O)	-0.3486	-0.3621	-0.3382	0.0104	0.0135	0.0119	0.0031
30(O)	-0.4049	-0.4082	-0.4017	0.0032	0.0033	0.0033	0.0001
51(C)	-0.0857	-0.0911	-0.0793	0.0065	0.0053	0.0059	-0.0011
55(O)	-0.2011	-0.211	-0.1905	0.0105	0.0099	0.0102	-0.0007

The toxicity of the identified degradation intermediates was computationally evaluated using the T.E.S.T (Toxicity Estimation Software Tool, version 5.1.2.0). The prediction endpoints included acute toxicity (96-hour LC50 for Fathead minnow, 48-hour LC_{50} for Daphnia magna, and 48-hour IGC_{50} for Tetrahymena pyriformis), as well as developmental toxicity and mutagenicity. The Consensus method was adopted as the primary prediction approach, except for the T. pyriformis IGC_{50} (48 h), for which the Nearest Neighbor method was employed. The detailed prediction results are presented in **Table S3**, where "N/A" denotes unavailable data. The acute toxicity was further classified into four hazard levels, namely "very toxic," "toxic," "harmful," and "not harmful," according to the criteria summarized in **Table S4**.

Table S3. Toxicity prediction of tetracycline hydrochloride degradation intermediates (Note: The acute toxicity endpoints are presented as logarithmic values.).

Samples	Fathead minnow LC_{50} (96 hr)	Daphnia magna LC_{50} (48 hr)	T. pyriformis IGC50 (48 h)	Developmental toxicity	Mutagenicity
TC	0.04576	0.7356	0.81291	1	0.6
P1	0.37291	0.64836	0.63749	1	0.19
P2	0.4183	1.19948	0.64444	N/A	0.49
P3	--	--	--	0.33	N/A
P4	1.88767	1.67006	0.92428	N/A	0.18
P5	0.90091	1.00903	0.89432	N/A	0.48
P6	2.73356	1.67062	1.44404	0.67	0.36
P7	2.72895	1.8515	--	0.67	0.32
P8	2.14139	1.91619	1.83059	0.33	0.32

Table S4. Acute toxicity prediction classification criteria.

toxicity	Very toxic	Toxic	Harmful	Not harmful
Log（concentration）	Log LC_{50}<0	0< Log LC_{50}<1	1< Log LC_{50}<2	Log LC_{50}>2

References

[1] M J Frisch, G W Trucks, H B Schlegel, et al. Gaussian 16 [Z]. Wallingford, CT; Gaussian, Inc. 2016

[2] P J Stephens, F J Devlin, C F Chabalowski, et al. Ab Initio Calculation of Vibrational Absorption and Circular Dichroism Spectra Using Density Functional Force Fields [J]. The Journal of Physical Chemistry, 1994, 98(45): 11623-7.

[3] S Grimme, J Antony, S Ehrlich, et al. A consistent and accurate ab initio parametrization of density functional dispersion correction (DFT-D) for the 94 elements H-Pu [J]. The Journal of Chemical Physics, 2010, 132(15): 154104.

[4] F Weigend. Accurate Coulomb-fitting basis sets for H to Rn [J]. Physical Chemistry Chemical Physics, 2006, 8(9): 1057–65.

[5] A V Marenich, C J Cramer, D G Truhlar. Universal Solvation Model Based on Solute Electron Density and on a Continuum Model of the Solvent Defined by the Bulk Dielectric Constant and Atomic Surface Tensions [J]. The Journal of Physical Chemistry B, 2009, 113(18): 6378–96.

[6] R G Parr, W Yang. Density Functional Approach to the Frontier-Electron Theory of Chemical Reactivity [J]. Journal of the American Chemical Society, 1984, 106(14): 4049–50.

[7] T Lu, F Chen. Multiwfn: A multifunctional wavefunction analyzer [J]. Journal of Computational Chemistry, 2012, 33(5): 580–92.

[8] W Humphrey, A Dalke, K Schulten. VMD: Visual Molecular Dynamics [J]. Journal of Molecular Graphics, 1996, 14(1): 33–8.

Disclaimer/Publisher's Note: The statements, opinions and data contained in all publications are solely those of the individual author(s) and contributor(s) and not of MDPI and/or the editor(s). MDPI and/or the editor(s) disclaim responsibility for any injury to people or property resulting from any ideas, methods, instructions or products referred to in the content.
